# A Saúde Cardiometabólica dos Adolescentes é Afetada por Períodos Prolongados de Inatividade?

**DOI:** 10.36660/abc.20210479

**Published:** 2021-08-09

**Affiliations:** 1 Universidade Federal de Pelotas PelotasRS Brasil Universidade Federal de Pelotas (UFPEL), Pelotas, RS – Brasil

**Keywords:** Doenças Cardiovasculares/fisiopatologia, Doenças Metabólicas, Atividade Física, Sedentarismo, Adolescente, Colesterol, Triglicérides, Índice de Massa Corpórea, Epidemiologia

A literatura atual sobre atividade física (AF) e comportamento sedentário (CS) destaca o efeito negativo de quantidades consideráveis de tempo gasto em atividades, tais como sentar, assistir TV, usar o computador e algumas atividades de trabalho e estudo, sobre a saúde cardiovascular.^1^ O CS pode ser definido como qualquer comportamento de vigília caracterizado por gasto energético ≤ 1,5 equivalentes metabólicos em uma postura sentada, reclinada ou deitada.^2^^,^^3^ Portanto, o CS não é a ausência ou um baixo nível de AF, ambos podem coexistir.^2^ Nesse sentido, uma revisão recente mostrou uma interação entre o CS e a AF, evidenciando que indivíduos com maior tempo de permanência em CS apresentam maior risco de mortalidade cardiovascular. No entanto, as estimativas têm sido menos consistentes em indivíduos com níveis mais elevados de AF.^4^

A literatura cita uma série de possíveis mecanismos para os efeitos do CS, independente da AF, sobre desfechos metabólicos e cardiovasculares. Um desses mecanismos é a diminuição da atividade enzimática responsável pela produção de HDL e a captura de triglicerídeos na corrente sanguínea, devido à inatividade sustentada na postura sentada, reclinada ou deitada.^1^ Nesse sentido, têm sido estudadas estratégias para reduzir o tempo de permanência em CS ou para interromper a inatividade sustentada. Algumas dessas abordagens se concentram em permanecer em pé durante algum tempo ou realizar um curto período de movimento entre os períodos de tempo sentado (breaks). Uma metanálise com adultos encontrou um efeito positivo dos breaks no CS em relação ao controle da adiposidade e glicemia.^5^ Além disso, um estudo experimental mostrou que pausas de 1 a 2 minutos em atividades sedentárias de trabalho a cada meia hora resultaram em declínios pequenos à moderados no colesterol total, triglicerídeos e glicemia de jejum.^6^

Enquanto a literatura sobre pausas foi desenvolvida focando principalmente na população adulta, investigando pausas nas atividades laborais sedentárias, estudos com crianças e adolescentes são escassos, especialmente em países de baixa e média renda. Além disso, a avaliação dos efeitos das pausas na saúde de adolescentes deve ser reforçada, visto que os riscos cardiometabólicos já estão presentes nessa idade,^7^^–^^10^ que também é marcada por atividades escolares sedentárias sustentadas. Diante desse cenário, Quirino et al.,^11^ em estudo publicado neste volume, verificaram a associação entre breaks no CS e o risco cardiometabólico em uma amostra de adolescentes. Este estudo transversal incluiu dados de 573 adolescentes de João Pessoa, PB, Brasil, e mediu objetivamente os breaks no CS utilizando acelerômetros. Os desfechos avaliados foram pressão arterial sistólica e diastólica, glicemia de jejum, colesterol total, triglicerídeos, HDL, LDL e índice de massa corporal (IMC).^11^ Os autores verificaram que um número maior de breaks no CS diminuiu o IMC em −0.102 kg/m. Não foram encontrados efeitos estatisticamente significativos para os demais desfechos. No entanto, a direção das associações foi no sentido de maior numero de breaks resultando em reduções nos desfechos negativos. A literatura com amostras de adultos encontrou associações com muitos desfechos, diferentemente dos resultados encontrados por Quirino et. al. Os autores trazem, como uma explicação para esse achado, as diferenças nos padrões de movimento de crianças e adolescentes. Crianças e adolescentes, em geral, têm um padrão de movimento com mais picos de AF de alta intensidade e janelas curtas de inatividade (tempo sedentário mantido por menos tempo).^12^^,^^13^

Este estudo levanta algumas questões para pesquisas futuras nesta área, por exemplo: Qual deve ser a duração dos braks no cs para obter um efeito positivo na saúde dos adolescentes? Os níveis de AF modulam os breaks no cs para esta população? Qual padrão de breaks poderia melhorar a saúde cardiometabólica (apenas permanecer em pé ou realizar alguns minutos de AF leve)? As respostas para essas perguntas poderão ajudar a planejar estratégias escolares para esse grupo populacional. Por último, mas não menos importante, é importante destacar que a atual pandemia de COVID-19 pode ter aumentado o tempo de permanência em CS na população geral,^14^^,^^15^ incluindo crianças e adolescentes. Dessa maneira, intervenções que aumentem o tempo de AF e o número de breaks no CS, que já deveriam ser incentivadas em situações normais, provavelmente tornam-se ainda mais relevantes neste cenário alarmante.
