# Supplementary material for: A Saúde Cardiometabólica dos Adolescentes é Afetada por Períodos Prolongados de Inatividade?
Source: Arq Bras Cardiol. 2021 Aug 9;117(2):363–4. [Article in Portuguese] doi: 10.36660/abc.20210479 (PMC8395811; doi:10.36660/abc.20210479)
Supplement: Translation

# Translation: Is Adolescents' Cardiometabolic Health Affected by Prolonged Periods of Inactivity?

**Affiliations:** 1 PelotasRS Brazil Universidade Federal de Pelotas (UFPEL), Pelotas, RS – Brazil

**Keywords:** Cardiovascular Diseases/physiopathology, Metabolic Diseases, Motor Activity, Sedentarism, Adolescent, Cholesterol, Triglycerides, Body Mass Index, Epidemiology

The current literature regarding physical activity (PA) and sedentary behavior (SB) highlights the negative effect of considerable amounts of time spent in activities such as sitting, TV viewing, computer use, and some work and study activities on cardiovascular health.^1^ SB may be defined as any waking behavior characterized by an energy expenditure ≤ 1.5 metabolic equivalents while in a sitting, reclining, or lying posture.^2^^,^^3^ Thus, SB is not the absence or low level of PA, but it can coexist with it.^2^ In this sense, a recent review showed an interaction between SB and PA, providing evidence that individuals with higher time spent in SB presented a higher risk of cardiovascular mortality. However, estimates have been less consistent in individuals with higher PA levels.^4^

The literature cites a series of possible mechanisms for SB effects, independent of PA, on metabolic and cardiovascular outcomes. One of these mechanisms is the decrease in enzymatic activity responsible for HDL production and the capture of triglycerides in the blood chain due to sustained inactivity in sitting, reclining, or lying posture.^1^ In this regard, strategies reducing the time spent in SB or interrupting sustained inactivity have been studied. Some of these approaches focus on standing up for a while or a short period of movement between periods of sitting time (breaks in SB). A meta-analysis with adults found a positive effect of breaks in SB on adiposity control and glycemia.^5^ In addition, an experimental study showed that 1-to-2–minute breaks in sedentary work activities every half an hour resulted in small-to-moderate declines in total cholesterol, triglycerides, and fasting blood glucose.^6^

While the literature about breaks was developed focusing mainly on adult populations and exploring interruptions in sedentary work activities, studies with children and adolescents are scarce, especially in low- and middle-income countries. Furthermore, the evaluation of the effects of breaks on adolescent health should be reinforced, given that cardiometabolic risks are already present at this age,^7^^–^^10^ which is also marked by sustained sedentary school activities. Faced with this scenario, Quirino et al.,^11^ in a study published in this volume, verified the association of breaks in SB on cardiometabolic risk in an adolescent sample. This cross-sectional study comprised data of 573 adolescents from João Pessoa, Paraíba, Brazil and objectively measured breaks in SB using accelerometers. Systolic and diastolic blood pressure, fasting glucose, total cholesterol, triglycerides, HDL, LDL, and body mass index (BMI) were the assessed outcomes.^11^ The authors found that a higher number of breaks in SB decreased BMI by −0.102 kg/m. Statistically significant effects for other outcomes were not found. However, the direction of associations was towards more breaks reducing negative outcomes. The literature with adult samples has found associations with many outcomes, different from the results found in this paper. The authors bring, as one explanation for this finding, the differences in movement patterns for children/adolescents. Children and adolescents, in general, have a movement pattern with more peaks of high-intensity PA and short inactivity windows (sedentary time sustained for less time).^12^^,^^13^

The study raises some future research questions for the area: How long should breaks in SB be in order to obtain a positive effect on adolescent health? Do PA levels modulate breaks in SB for this population? Which is the ideal pattern of breaks supposed to improve cardiometabolic health (only standing up or a few minutes of light PA)? Answering these questions may help to plan school-based strategies for this population group. Last but not least, it is important to highlight that the current COVID-19 pandemic may have increased the time spent in SB by the general population,^14^^,^^15^ including children and adolescents. Thus, interventions increasing the time spent in PA and breaks in SB, which should be encouraged in normal situations, are likely even more relevant in this alarming scenario.
